# Advances in Hydrodechlorination Technologies for Diclofenac Removal from Aqueous Systems

**DOI:** 10.3390/molecules30163332

**Published:** 2025-08-09

**Authors:** Cristian Castillo, Jorge A. Mora, Maria H. Brijaldo

**Affiliations:** 1 Centro de Química Estrutural, Institute of Molecular Sciences, Departamento de Engenharia Química, Instituto Superior Técnico, Universidade de Lisboa, 1049-001 Lisboa, Portugal; cristian.navas@tecnico.ulisboa.pt; 2 Grupo de Investigación de Farmacia y Medio Ambiente (FARQUIMA), Universidad Pedagógica y Tecnológica de Colombia, Avenida Central del Norte, vía Paipa, Tunja 150003, Boyacá, Colombia; jorgearmando.mora@uptc.edu.co

**Keywords:** diclofenac, hydrodechlorination, catalysts, aqueous systems

## Abstract

This review article describes the most recent studies carried out on the catalytic hydrodechlorination (HDC) of Diclofenac (DFC). In this context, the most commonly employed catalytic materials for the removal of DFC from aqueous matrices are reviewed, along with their main performance outcomes. Various strategies for the HDC of DFC are discussed, including conventional approaches that rely on molecular hydrogen as the electron donor, as well as emerging alternatives based on biocatalytic and electrocatalytic processes. Additionally, the optimized reaction conditions for each catalytic system are discussed, along with relevant kinetic models and mechanistic insights that contribute to a deeper understanding of the HDC of DFC. Future perspectives on the use of catalysts with alternative properties for DFC removal via HDC are also discussed, aiming to highlight potential applications in wastewater treatment and the broader field of heterogeneous catalysis.

## 1. Introduction

The occurrence of organic micropollutants, particularly active pharmaceutical ingredients (APIs), in aquatic environments has become a pressing environmental concern due to their widespread use, persistence, and resistance to conventional wastewater treatment technologies. Among these, non-steroidal anti-inflammatory drugs (NSAIDs) such as DFC are frequently detected in surface and effluent waters, primarily as a result of incomplete biodegradation in municipal wastewater treatment plants (WWTPs) [1,2,3]. APIs can enter aquatic systems through various pathways, including human and veterinary excretion or the direct disposal of unused medications, often appearing in their parent form or as bioactive metabolites [4]. Although typically present at trace levels (ng·L^−1^ to mg·L^−1^), these compounds pose considerable ecological risks. In particular, DFC has been shown to induce hepatic and renal toxicity in fish, along with histopathological damage to gill tissues, at concentrations as low as 1 μg·L^−1^ [5,6,7,8]. The environmental relevance of DFC is further emphasized by its high global usage, estimated at approximately 940 tons per year, and its recurrent detection in WWTPs effluents, surface waters, and even drinking water sources [9,10].

Various strategies have been investigated to mitigate the environmental impact of DFC contamination. Adsorptive removal using carbon-based materials and advanced oxidation processes (AOPs)—such as the Fenton reaction and ultraviolet (UV) irradiation in the presence of H_2_O_2_ or O_3_—have been widely explored to achieve complete degradation of DFC [11,12,13]. For instance, Jiang et al. [14] demonstrated visible-light-promoted peroxymonosulfate activation for acetaminophen (ACE) degradation, revealing the central role of photogenerated holes in the oxidation process. Similarly, Xie et al. [15] achieved efficient ciprofloxacin (CPFX) removal using iron-doped carbon quantum dots, where the network capture effect significantly enhanced the activation of peroxymonosulfate, as supported by computational calculations. These studies underscore the environmental urgency to develop complementary technologies such as HDC for addressing APIs like DFC in aquatic systems.

However, these oxidative treatments often lead to the generation of toxic transformation products and exhibit limited effectiveness in cleaving stable carbon–halogen bonds, resulting in persistent hazardous by-products. Other approaches, including adsorption on activated carbon and membrane filtration, can remove halogenated compounds to some extent, yet they frequently fail to fully address residual toxicity and compound stability issues [11,12,13,16,17,18,19]. Biological methods, such as the use of organochlorine-respiring bacteria, have demonstrated the ability to dechlorinate DFC, but the slow kinetics and operational impracticality restrict their applicability at large scales [20]. Gamma irradiation, although effective, is constrained by high operational costs and practical limitations [21].

In recent years, liquid-phase catalytic HDC has attracted considerable attention as an effective technique for the reductive removal of chlorinated organic pollutants. Pd-supported catalysts have demonstrated high catalytic activity in HDC processes, representing a promising approach for the degradation of chlorinated compounds [13]. Recent developments indicate that bimetallic catalysts, such as Pd/Au, exhibit enhanced catalytic performance compared to monometallic Pd, owing to synergistic effects that improve both activity and stability [20,21]. These enhancements are attributed to geometric and electronic interactions between the metals, which inhibit particle aggregation and increase catalytic efficiency [22]. Despite these advances, comprehensive studies on the liquid-phase catalytic HDC of DFC using supported noble metals remain limited, and the potential toxicity of the dechlorinated products warrants further investigation to fully evaluate their environmental and health impacts [22,23].

One of the approaches currently considered is the biocatalytic HDC of DFC by using Pd nanoparticles (NPs) synthesized by microorganisms as a cost-effective and eco-friendly method. Microbial reduction produces biogenic Pd NPs that are well-dispersed and stabilized on cell surfaces or within the extracellular polymeric substance (EPS) matrix. Compared to chemically or physically synthesized Pd NPs, biosynthesized ones exhibit enhanced stability and catalytic activity. Studies have shown their effectiveness in catalyzing DFC dechlorination under environmental conditions, using electron donors such as formate, glucose, acetate, and hydrogen gas (H_2_), with H_2_ being the most efficient [24,25,26]. Nevertheless, the low H_2_ solubility in water limits its long-term supply in catalytic processes. To address this, membrane biofilm reactors (MBfRs) have been developed to deliver pressurized H_2_ via bubble-free gas-permeable membranes [27]. In situ biosynthesis of Pd NPs in denitrifying biofilms within MBfRs allows immediate activation by hydrogen donors, enhancing continuous DFC dechlorination.

As an emergent technique that is considered a new approach to HDC processes, electrocatalytic hydrodechlorination (ECHDC) is a promising method for degrading chlorinated organic pollutants due to its mild conditions and high efficiency [8]. Noble metals exhibit excellent catalytic activity for ECHDC, but, to reduce costs and enhance performance, these metals are often supported on high-surface-area materials such as Ni foam, Ti mesh, and graphene [28,29,30,31,32]. However, challenges remain in treating low-concentration pollutants, as limited mass transfer leads to long reaction times and high energy consumption [33]. Three-dimensional (3D) electrodes, composed of particle electrodes between 2D electrodes, improve pollutant contact, enhance electrolyte convection, and accelerate reaction rates, making them suitable for low-concentration treatments [34]. However, ECHDC on amorphous catalysts has been scarcely explored in the current literature [35].

Based on the aspects mentioned above, this work aims to critically analyze recent advances in the catalytic HDC of DFC, focusing on commonly used catalytic materials, optimized reaction conditions, and the associated kinetic models and mechanistic insights. Additionally, emerging biocatalytic and electrocatalytic strategies are discussed, along with future perspectives on the development of novel catalysts for efficient DFC removal from aqueous environments.

## 2. Current Studies Covering Aqueous-Phase HDC of DFC

To provide a critical perspective, the studies discussed in this section have been grouped thematically based on catalyst type and performance trends, rather than strictly chronologically. This approach facilitates the identification of patterns, consistencies, and discrepancies across the literature.

Since 2012, extensive efforts have focused on studying the HDC process to reduce DFC concentrations in water by transforming it into less toxic chemical compounds. Research has evaluated various aspects of catalysts, including metal phase, support type, and synthesis method. Structural and catalytic properties vary with preparation, with impregnation and deposition–precipitation being the most used techniques [36]. Reaction conditions, mechanistic insights, and kinetic modeling regarding the HDC of DFC have also been investigated. Complementing laboratory-scale experiments, studies assessing the toxicity of post-treated DFC solutions and evaluating the efficacy of the HDC process in removing DFC from environmentally relevant water matrices have also been reported.

While DFC has been widely employed as a representative model for studying HDC due to its environmental relevance and structural recalcitrance, recent studies have also reported the application of HDC techniques to other APIs, such as chloramphenicol (CAP), triclosan (TCL), and sertraline (SRT). For instance, Pd-based electrocatalytic systems have shown promising results in the reduction of CAP under mild aqueous conditions, significantly improving catalyst dispersion and stability by using NiFe-MOF-based supports and achieving efficient detoxification as confirmed by ecological structure–activity relationship (ECOSAR) models and density functional theory (DFT) studies [37,38,39,40,41,42,43,44]. TCL, another highly persistent and toxic compound, has been effectively treated using both biocatalytic and electrocatalytic HDC. Microbial Pd NPs have demonstrated simultaneous dechlorination and oxidation of TCL, reducing cytotoxicity by over 30%, while electrocatalytic approaches have enhanced atomic hydrogen production and achieved >80% toxicity reduction in treated water [45,46,47,48,49]. These findings confirm that the scope of HDC extends beyond DFC, providing strong evidence of its versatility for treating a broader spectrum of halogenated APIs. However, the limited number of studies for each compound and the lack of standardized methodologies support the decision to focus this review on DFC, which currently offers the most comprehensive and consistent dataset for critical comparison.

**Figure 1:**
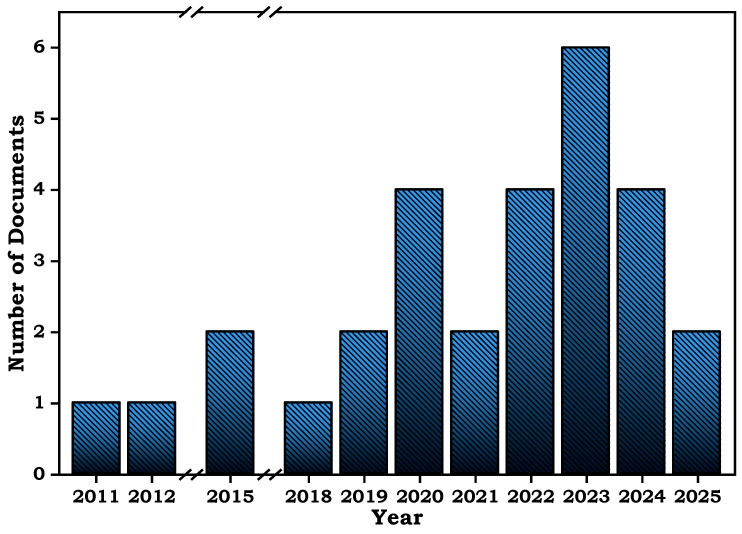
Number of documents published in the period 2011–2025 regarding the DFC HDC. (Bibliometric analysis was carried out in Web of Science by using the Keywords “Hydrodechlorination” and “Diclofenac”).

Figure 1 illustrates the scientific documents published from 2011 to 2025 related to the HDC of DFC that have focused on different subject areas such as chemistry, chemical engineering, and materials science. It is important to mention that different approaches have been employed to address the HDC of DFC by using the traditional one, which involves the use of hydrogen as an electron donor, and other pathways that are focused on the use of biocatalytic and electrocatalytic procedures. Some of the research articles (Table 1) published between 2011 and 2025 were systematically selected for this review. While this compilation does not claim to be exhaustive, it encompasses the most representative studies addressing the HDC of DFC, chosen based on their relevance, methodological clarity, and contribution to understanding catalyst performance. Although several of these articles were published over a decade ago, they remain fundamental to the historical evolution and current understanding of HDC processes.

Wu et al. [36] synthesized Pd-catalysts (~2.0 wt%) by the deposition–precipitation method (*dp-*) and various supports (CeO_2_, Al_2_O_3_, activated carbon (AC), and SiO_2_) in order to test their influence on the catalytic activity during the HDC of DFC. For comparison purposes, the Pd/CeO_2_ catalyst was obtained by using the impregnation method, and it was denoted as *im*-Pd/CeO_2_. Subsequently, all catalysts were characterized by X-ray Diffraction (XRD), Transmission Electron Microscopy (TEM), N_2_ physisorption, X-ray Photoelectron Spectroscopy (XPS), CO chemisorption, and Zeta Potential. In addition, these materials were evaluated in the HDC of DFC, employing 20 mg of catalyst and a DFC solution adjusted to pH 9.0, with initial concentrations varied from 0.06 to 0.24 mM and a H_2_ flow of 250 mL·min^−1^. The *dp*-Pd(1.7)/CeO_2_ catalyst exhibited the best catalytic performance, achieving 98% of DFC conversion after 30 min of reaction, while the *im*-Pd/CeO_2_ catalyst showed a lower performance (DFC conversion of 76%). The best behavior exhibited by *dp*-Pd(1.7)/CeO_2_ catalyst was attributed to the higher Pd dispersion, smaller Pd particle, and more cationized Pd species due to the existence of stronger metal–support interaction. Due to these properties, the *dp*-Pd(1.7)/CeO_2_ catalyst lost 75% of its activity after five consecutive reaction cycles due to its deactivation. These findings align with other studies that emphasize the critical role of support material and metal dispersion.

Wang et al. [50] developed Au–Pd bimetallic NPs with a core–shell configuration, where Au serves as the core and Pd as the shell. These Au–Pd core–shell NPs were characterized by scanning electron microscopy (SEM) and high-resolution TEM (HRTEM), and they were subsequently employed for the first time to explore the HDC of DFC under aqueous conditions at room temperature. The findings revealed that nearly 100% of DFC (30 mg·L^−1^, 50 mL, pH = 7) was dechlorinated within 4.5 h using 10 mL of 56 mg·L^−1^ Au–Pd core–shell NPs. This performance was likely due to a synergistic interaction between the two metals, potentially arising from geometric effects, electronic effects, or a combination of both. These results are consistent with [36], reinforcing that nanostructuring and bimetallic interfaces boost Pd catalytic activity.

On the other hand, Nieto-Sandoval et al. [51] evaluated the commercial catalyst Pd/Al_2_O_3_ (1 wt%) (Alfa Aesar) due to its physicochemical properties such as a mean diameter of 24 μm, specific area of 270 m^2^.g^−1^, and a Pd^0^/Pd^n+^ molar ratio of 1.04. The catalyst was completely characterized by N_2_ physisorption, total reflection X-ray fluorescence (TXRF), and XPS. The HDC tests were carried out by using [DFC]_0_ = 0.068 mM, catalyst concentrations from 0.1 to 2.0 g.L^−1^, temperatures in the range of 17–35 °C, and stirring velocities of 600–1000 rpm. Complete DFC degradation was achieved after 20 min of reaction, observing APA (2-anilinophenylacetate) as the only product formed, employing ambient conditions (25 °C, 1 atm) with [Pd/Al_2_O_3_]_0_ = 0.5 g.L^−1^ and an H_2_ flow rate of 50 N·mL·min^−1^. Remarkably, the catalyst exhibited a reasonable stability upon three successive uses, achieving complete DFC degradation and obtaining APA as the final product after 30 min of reaction. Furthermore, trace amounts of 2-cyclohexylaminophenylacetate (CPA) were detected under several conditions. This last product was not previously detected by Wu et al. [36], demonstrating the high hydrogenation activity of the Pd/Al_2_O_3_ commercial catalyst.

Subsequently, Nieto-Sandoval et al. [52] again tested the commercial catalyst Pd/Al_2_O_3_ (1 wt%) during HDC of different chlorinated micropollutants such as the antibiotic CAP, the anti-inflammatory DFC, the antibacterial agent TCL, and the antidepressant SRT, which are commonly found in the source waters of drinking water treatment plants (DWTPs). The complete degradation of the isolated micropollutants (3 mg·L^−1^) was achieved after 1 h of reaction using a [Pd/Al_2_O_3_]_0_ = 0.25 g.L^−1^ and a H_2_ flow rate of 50 N·mL·min^−1^. These studies collectively confirm the effectiveness and reproducibility of Pd/Al_2_O_3_ under ambient conditions.

Kowalewski et al. [53] investigated the effect of unimodality and bimodality of Pd NPs on the catalytic activity of Pd/SiO_2_ during the HDC of DFC. For this purpose, different Pd/SiO_2_ catalysts were synthesized employing ion exchange of the hydroxyl group of silica (1.1 wt% Pd/SiO_2_(s)) and the incipient wetness impregnation methods by using two different precursors: (CH_3_COO)_2_Pd and PdCl_2_ for 2.0 wt% Pd/SiO_2_(bg) and 2.0 wt% Pd/SiO_2_(bim) preparation, respectively. Then, these catalytic materials were characterized by N_2_ physisorption, temperature-programmed hydride decomposition (TPHD), and TEM. Finally, the HDC reactions were carried out in a batch reactor by using the following operating conditions: atmospheric pressure, 25 °C, 1 g of catalyst, [DFC]_0_ = 240 μM, and H_2_ flow of 1 mL·min^−1^. After 90 min of HDC reaction, the catalytic activity decreased in the following order: Pd/SiO_2_(bim) > Pd/SiO_2_(bg) > Pd/SiO_2_(s).

The complete degradation of DFC was achieved, and this result was explained due to the coexistence of smaller and bigger Pd NPs, which favored the activation of DFC and H_2_, avoiding the deactivation of the catalyst due to the lack of H_2_, which is necessary for the activation of the C-Cl bond. In this way, they concluded that Pd NPs with different average particle sizes and distribution supported on SiO_2_ are potential catalytic materials to be tested in DFC degradation by HDC. As such, this work introduces the concept of particle size distribution as a performance factor, contrasting with monodispersed systems such as the aforementioned systems in the previous paragraphs.

Likewise, Zawadzki et al. [54] obtained two 1 wt% Pd-loaded BEA zeolites (Si/Al = 19 and 1300) by using a two-step post-synthesis method (PdSiBEA) and the wet impregnation method (PdHAlBEA). These materials were evaluated in the HDC of DFC and compared with the catalysts Pd/SiO_2_(s) and Pd/Al_2_O_3_, which were obtained, respectively, by the ion exchange between the hydroxyl group of silica and [Pd(NH_3_)_4_](NO_3_)_2_ and the incipient wetness impregnation methods. All catalysts were characterized by N_2_ physisorption, CO chemisorption, and TEM. Subsequently, the catalytic performance of this group of catalysts was investigated by employing a batch reactor at mild conditions (atmospheric pressure, 30 °C) and [DFC]_0_ = 240 µM. DFC conversions between 88 and 100% were achieved by using the zeolite-based catalysts after 20 min of reaction. The three-dimensional zeolite structure stabilized small and large Pd particles, enhancing performance. These findings are consistent with [36] and [53], which underline the role of support porosity and metal dispersion in catalyst design.

Bendová et al. [56] investigated a Raney Al–Ni material, comprising 62% Ni_2_Al_3_ and 38% NiAl_3_ crystalline phases, as a highly effective catalyst for the hydrodehalogenation (HDH) of DFC. Its performance was evaluated in both model aqueous solutions and real hospital wastewater. In model conditions, complete HDC of a 2 mM DFC solution (0.59 g.L^−1^) was achieved within 50 min at room temperature and ambient pressure, employing only 9.7 g.L^−1^ KOH and 1.65 g.L^−1^ of Raney Al–Ni. The transformation led to the formation of APA as the main dechlorinated product. During the reaction, aluminum dissolution was observed alongside the complete consumption of the NiAl_3_ phase and partial depletion of Ni_2_Al_3_. To assess the broader applicability of the catalyst, HDH activity was also tested using a mixture of DFC and other commonly used halogenated aromatic and heterocyclic biocides in model solutions.

The results confirmed consistently high dehalogenation efficiency across all tested compounds. Notably, the robustness of the Al–Ni-based system was further validated under realistic conditions, achieving effective DFC removal from hospital wastewater characterized by high chloride and nitrate levels.

Taking into account that most of the studies regarding the HDC of DFC were conducted in batch reactors, Nieto-Sandoval et al. [57] developed and applied a Pd-based catalytic membrane reactor (Pd/CMR) for continuous-flow HDC. For this purpose, a cylindrical, inert, porous alumina membrane was functionalized on its outer surface with well-dispersed palladium NPs (0.2 wt.%).

The resulting Pd/CMR was thoroughly characterized using multiple analytical techniques such as N_2_ physisorption, inductively coupled plasma mass spectrometry (ICP-MS), elemental analyses, TEM, XPS, XRD, and thermogravimetric analysis (TGA). HDC experiments were conducted in a double-jacketed glass tube reactor operated in continuous up-flow mode. The system was fed with an aqueous influent containing 100 μg·L^−1^ of DFC at a flow rate of 0.2 mL·min^−1^ and 25 °C. The catalytic performance of the Pd/CMR was assessed under long-term continuous operation for up to 200 h on stream. The effects of key operational parameters, specifically the initial DFC concentration (ranging from 100 to 500 μg·L^−1^) and feed flow rate (0.1 and 0.2 mL·min^−1^), were systematically investigated. As the main result, the concentration of DFC in the reactor effluent remained stable throughout the operation period, corresponding to a consistent conversion of approximately 64% over 120 h on stream. Catalyst stability was assessed under these conditions to monitor potential deactivation from the onset of the reaction. Although complete removal of DFC and its chlorinated intermediate was not achieved under these settings, further optimization of operating parameters may enable full conversion.

The catalytic performance differences observed in the literature for DFC HDC are primarily driven by the physicochemical properties of the active phase, the nature of the support, and the preparation method. Catalysts supported on CeO_2_ [36] and BEA zeolites [54] consistently exhibit superior activity due to their ability to promote strong metal–support interactions and stabilize highly dispersed Pd NPs. These features increase the number of active sites and facilitate H_2_ activation. In contrast, catalysts supported on inert oxides such as SiO_2_ [53] show limited activity unless engineered with bimodal Pd distributions, which enable cooperative activation of DFC and H_2_. Bimetallic systems such as Au–Pd [50] or Pd–Ni [58] display enhanced performance through electronic synergism, modifying the *d*-band center of Pd and improving its affinity for C–Cl bond cleavage.

Finally, the catalyst synthesis method plays a crucial role: deposition–precipitation typically yields smaller, more uniform Pd particles compared to impregnation, correlating with higher DFC conversion rates [36]. These structure–activity relationships explain the wide variability in HDC performance and underscore the importance of tailoring both composition and nanostructure for optimal catalytic function. This thematic organization also allows for clearer comparison of consistent trends across studies, and highlights discrepancies that often stem from differences in support properties, metal particle dispersion, or experimental configurations.

Overall, current studies provide a solid foundation for the aqueous-phase HDC of DFC, setting the stage for a detailed exploration of the kinetic models and mechanistic insights that underpin these catalytic processes.

## 3. HDC of DFC: Kinetic Models and Mechanistic Insights

In general, for a heterogeneous reaction, and specifically for the HDC of DFC, the DFC adsorption onto the catalytic surface is a critical initial step. Table 2 shows the kinetic parameters of the HDC of DFC by using different catalysts. Usually, to assess the impact of DFC adsorption, different initial concentrations of DFC are employed. For example, in the study carried out by Wu et al. [36], the DFC concentrations were varied from 0.06 to 0.24 mM, and, in this way, the initial catalytic activity increased from 3.9 to 11.6 mM.g cat^−1^.h^−1^, indicating that the catalysis is primarily controlled by the adsorption process. This adsorption-driven mechanism can be further corroborated by applying the experimental data to the Langmuir–Hinshelwood model [59]:

(1) $$r_{0}=k\theta_{s}=k\frac{bC_{0}}{1\;+\;bC_{0}}$$

(2) $$\frac{1}{r_{0}}=\frac{1}{kbC_{0}}+\frac{1}{k}$$

where $r_{0}$ is the initial reaction rate, $C_{0}$ is the initial reactant concentration, $\theta_{s}$ is the surface coverage of Diclofenac on the catalyst surface, k is the reaction rate constant, and b is the adsorption constant. Commonly, the dependence of $r_{0}$ on $C_{0}$ is verified by obtaining the $R^{2}$ value from a linear plot of $\frac{1}{C_{0}}$ versus $\frac{1}{r_{0}}$. In the specific case of the HDC of DFC carried out by using the *dp*-Pd(1.7)/CeO_2_ catalyst, $R^{2}$ > 0.99, the results indicated that the catalytic HDC of DFC could be accurately described by the Langmuir–Hinshelwood model, confirming that the reaction mechanism is controlled by adsorption. On the other hand, when the HDC of DFC using *dp*-Pd/CeO_2_ catalysts is conducted, the catalytic activity correlates with the Pd content, as exposed metallic Pd atoms act as the active sites. In this way, by increasing the Pd loading (from 0.45 to 2.6 wt%), the initial reaction rate is enhanced (from 3.5 to 10.5 mM.g cat^−1^.h^−1^) due to a higher number of exposed Pd sites. Analysis of turnover frequency (TOF) values indicated that while the TOF initially decreased with increasing Pd particle size, it then stabilized. This trend suggests that C–Cl bond activation is more critical than H_2_ activation in the HDC process, as higher Pd loading results in larger particles and fewer cationic Pd species [60]. It is important to mention that this kinetic model is applied even in HDC processes carried out by using bio-Pd-based catalysts [24,26,55].

Wu et al. [36] reported the possible hydrogenation of APA as the dechlorinated product of HDC. They indicated as main results that, after two h of reaction, the concentration of APA remained almost unchanged, and no additional products were detected through HPLC analysis. Therefore, the catalytic HDC of DFC towards APA may proceed through stepwise and/or concerted pathways, and, subsequently, the proposed dechlorination mechanism could be validated by fitting the kinetic data using the following equations [61]:

(3) $$C_{DFC}=C_{DFC}^{0}e^{\;-\;(k_{1}+k_{2})t}$$

(4) $$C_{Cl-APA}=\frac{k_{1}C_{DFC}^{0}}{k_{3}-k_{1}-k_{2}}(e^{-(k_{1}+k_{2})t}-e^{-k_{3}t})$$

(5) $$C_{APA}=\frac{k_{1}k_{3}C_{DFC}^{0}}{k_{3}-k_{1}-k_{2}}\left(\frac{-1}{k_{1}+k_{2}}\left(e^{-\left(k_{1}+k_{2}\right)t}-1\right)+\frac{1}{k_{3}}\left(e^{-k_{3}t}-1\right)\right)-\frac{k_{2}C_{DFC}^{0}}{k_{1}+k_{2}}\left(e^{-\left(k_{1}+k_{2}\right)t}-1\right)$$

where $C_{DFC}^{0}$ is the initial DFC concentration, $C_{DFC}$, $C_{Cl\;-\;APA}$, and $C_{APA}$ are concentrations of DFC, Cl-APA (2-(2-chloroanilino)-phenylacetate), and APA, respectively, at reaction time t. $k_{1}$, $k_{2}$, and $k_{3}$ are the rate constants of DFC, Cl-APA, and APA, respectively. The HDC of DFC on *dp*-Pd(1.7)/CeO_2_ revealed that the intermediate APA was detected, confirming a stepwise reaction pathway, while high rate constants ($k_{2}$) also supported a concerted pathway, indicating a combined mechanism. The *dp*-Pd(1.7)/CeO_2_ catalyst showed higher catalytic activity compared to the other supported catalysts based on Pd, with a notably higher $\frac{k_{2}}{k_{1}}$ ratio of 4:3. This increased ratio is likely due to cationic Pd species that enhance C–Cl bond activation. As Pd loading increased, the $\frac{k_{2}}{k_{1}}$ ratio rose, potentially because larger Pd particles provided more planar surface area, facilitating the concerted dechlorination pathway.

Likewise, Wang et al. [50] suggested that the HDC of DFC by using Au–Pd core–shell NPs occurs by stepwise and/or concerted mechanisms. However, they evaluated the variation in DFC concentration during HDC by fitting the first-order reaction and second-order reaction described through Equations (6) and (7). The kinetics of first-order have also been employed by other authors in the HDC of DFC, as shown in the following Equations [54]:

(6) $$C_{DFC}=C_{DFC}^{0}e^{-k_{1}t}$$

(7) $$C_{DFC}=C_{DFC}^{0}e^{-k_{1}t}+(1-C_{DFC}^{0})e^{-k_{2}t}$$

where $C_{DFC}^{0}$ is the initial DFC concentration, $C_{DFC}$ is the concentration of DFC at reaction time t, $k_{1}$ is the reaction rate constant of the first step in the stepwise pathway, and $k_{2}$ is the reaction rate constant of the concerted pathway. In this way, they found that the second-order model fits better because it has the higher correlation value ($R^{2}\;$= 0.9970), suggesting that the degradation of DFC catalyzed by Au–Pd core–shell structures involves multiple chemical reactions. The fact that $k_{2}$ is three times greater than $k_{1}$ indicates that the reaction rate described by the stepwise pathway predominantly determines the overall degradation rate. For simplicity, in subsequent analyses, the first-order reaction model was used to describe the DFC concentration trend, showing that the Au–Pd core–shell NPs exhibit the highest reaction rate constants, facilitating faster DFC degradation, approximately 46 times faster than that catalyzed by simple Pd NPs at room temperature.

On the other hand, Nieto-Sandoval et al. [51] also carried out a kinetic model of the HDC of DFC by using the Pd/Al_2_O_3_ commercial catalyst. Although it was possible to detect CPA under several HDC conditions, it was not included in the kinetic equations. Considering that the process occurs under kinetic control, with a constant catalyst concentration and an excess of hydrogen (H_2_) continuously supplied during the experiments, the following kinetic equations were proposed based on pseudo-first-order kinetics with respect to the reactants:

(8) $$r_{DFC}=\;-\;\frac{dC_{DFC}}{dt}=k_{1}C_{DFC}$$

(9) $$r_{Cl-APA}=\frac{dC_{Cl-APA}}{dt}=k_{2}C_{DFC}-k_{3}C_{Cl-APA}$$

(10) $$C_{APA}=\frac{dC_{APA}}{dt}=k_{3}C_{Cl-APA}$$

where $C_{DFC}$, $C_{Cl\;-\;APA}$, and $C_{APA}$ represent the concentrations of DFC, Cl-APA, and APA in solution (mM) and $k_{1}$, $k_{2}$, and $k_{3}$ are the apparent first-order rate constants. Given that DFC degradation is primarily driven by HDC but also includes adsorption, the rate constant $k_{1}$ for DFC removal involves both the HDC and adsorption effects. Therefore, the rate constant $k_{2}$, which only takes into account the HDC activity, was employed to model the generation of Cl-APA. The integrated equations were obtained by using $t=0,C_{DFC}=C_{DFC}^{0},C_{Cl\;-\;APA}=C_{APA}=0$ as initial conditions. These Equations (11) and (12) are shown below; nevertheless, the equation related to the variation in the DFC concentration was aforementioned (Equation (6)) and employed in the research carried out by Wang et al. [50]. In order to adjust the adsorption effect, the initial concentration of DFC available for the formation of Cl-APA and APA was represented as $C_{DFC_{0}}^{\prime }$.

(11) $$C_{Cl-APA}=\left(\frac{k_{2}C_{DFC_{0}}^{\prime }}{k_{3}-k_{2}}\right)\left(e^{-k_{2}t}-e^{-k_{3}t}\right)$$

(12) $$C_{APA}=\frac{C_{DFC_{0}}^{\prime }(1-\left(k_{3}e^{-k_{2}t}\right)-(k_{2}e^{-k_{3}t}))}{k_{3}-k_{2}}$$

After fitting all experimental data, it was observed that the rate constant $k_{1}$, which includes both HDC and adsorption effects, exhibited higher values than $k_{2}$, which only takes HDC into account. However, as the temperature increased, the differences between these constants became less significant, likely due to the reduced contribution of adsorption at higher temperatures. As aforementioned, Wu et al. [36] reported comparable rate constant values for the HDC of DFC by using the *dp*-Pd(1.7)/CeO_2_ catalyst under ambient conditions. In their work, the overall rate constant for the removal of the drug was the sum of $k_{1}$ (0.025 min^−1^) and $k_{2}$ (0.108 min^−1^), resulting in a value similar to that obtained by Nieto-Sandoval et al. [51]. Finally, the apparent activation energy ($E_{a}$) for the HDC of DFC was determined using the Arrhenius equation. For the removal of the drug ($k_{1}$), the activation energy was found to be 43 kJ.mol^−1^. For $k_{2}$, where only the HDC process is considered, the $E_{a}$ was 51 kJ.mol^−1^. These kinetic parameters were recalculated for the HDC of CAP, TCL, DFC, and SRT in later studies carried out by Nieto-Sandoval et al., obtaining similar values [52]. As new findings, they noticed that the reactivity of micropollutants toward HDC decreased in the following order: CAP > TCL > DFC > SRT. This trend was attributed to the number of available positions on the molecule where nucleophilic substitution by hydrogen can occur. Prior to this process, hydrogen molecules first undergo dissociative adsorption on the Pd surface [62,63].

**Figure 2:**
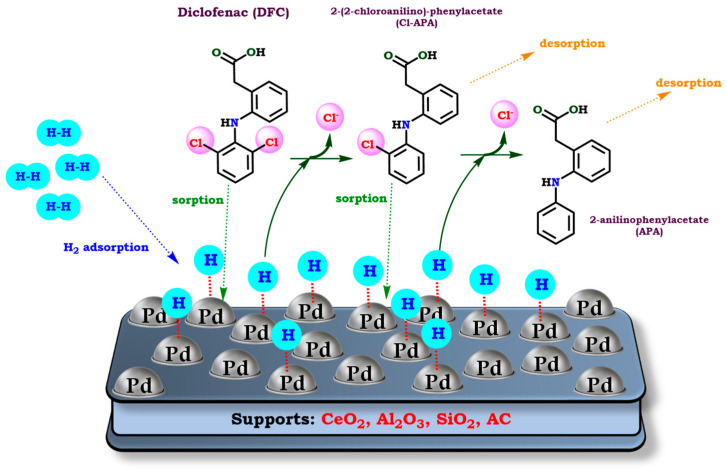
Proposed mechanism for the HDC of DFC over Pd-based catalysts. Adapted from Wu et. al, Wang et. al, Nieto-Sandoval et al., and De Corte et al. Reprinted with permission from Ref. [26] Copyright 2012 Elsevier. Ref. [36] Copyright 2015 Royal Society of Chemistry. Ref. [50] Copyright 2015 Taylor & Francis. Ref. [52] Copyright 2019 Elsevier.

According to all the information previously discussed, it is possible to illustrate in Figure 2 the approximated reaction mechanism for the HDC of DFC over Pd-based catalysts. Therefore, the reaction starts with the adsorption of hydrogen molecules over the Pd surface through a dissociative configuration [62,63]. After DFC is adsorbed onto the activated catalyst surface, the reaction could continue by two different pathways [26,51,52,55]. The first one consists of two successive hydrogenolysis reactions, resulting in the sequential formation of Cl-APA and APA. The subsequent product is the completely dechlorinated compound obtained after the HDC of DFC. In this way, APA is eventually desorbed from the catalyst surface. On the other hand, the mechanism could proceed by a concerted pathway, where DFC is directly converted to APA. Finally, under several conditions, it is possible to obtain CPA [51] as a result of the complete hydrogenation of the phenyl group in APA.

Kinetic studies have mainly been performed for the HDC of DFC in batch reactors, but few of the models have been developed to understand the kinetics of the HDC of DFC in continuous-flow reactors. Nieto-Sandoval et al. [57] addressed this approach, taking into account that the concentration of micropollutants in DWTPs can exhibit significant fluctuations. Therefore, they evaluated the influence of the initial pollutant concentration on the performance of the Pd/CMR system as an emergency technology for the HDC treatment of real water effluents. For this purpose, the DFC concentration was varied between 100 and 500 μg·L^−1^ (0.00034 to 0.00169 mmol.L^−1^). All tested concentrations yielded comparable conversion values, ranging from 58% to 64%. The consistent high activity of the Pd/CMR system, regardless of pollutant concentration, underscores its suitability for use as a polishing step in DWTPs. Based on these findings, it can be inferred that the HDC reaction follows first-order kinetics, as DFC conversion remained independent of its initial concentration. This observation is consistent with previous reports on HDC processes [64,65]. Given that the reaction operates under kinetic control and the hydrogen concentration was maintained constant throughout the experiments, an apparent pseudo-first-order kinetic model was applied to determine the corresponding HDC rate constants, as follows:

(13) $$\frac{W}{F_{DFC,\;0}}=\frac{W}{{Q\cdot C}_{DFC,\;0}}=\int_{0}^{X_{DFC}}\frac{dX_{DFC}}{k\cdot C_{DCF,0}\cdot (1\;-\;X_{DFC})}$$

where W represents the catalyst weight (g), Q is the feed flow rate (mL·min^−1^), $X_{DFC}$ denotes the conversion of DFC, and k is the apparent pseudo-first-order rate constant (mL·g cat^−1^.min^−1^). The aforementioned equation provides an excellent fit to the experimental data obtained across the range of initial DFC concentrations tested. In this way, the resulting HDC rate constant was determined to be 1.66 × 10^−2^ mL·g cat^−1^.min^−1^.

Finally, it is important to mention that a thorough understanding of the kinetic models and mechanistic pathways involved in the HDC of DFC is crucial for optimizing treatment efficiency, which naturally leads to evaluating the toxicity of post-treated DFC solutions to ensure environmental safety.

## 4. Toxicity Evaluation of Post-Treated DFC Solutions by HDC

Evaluating the toxicity of DFC solutions treated by HDC is essential to ensure that the dechlorinated products do not retain residual toxicity harmful to aquatic organisms such as *Daphnia magna* and *Vibrio fischeri*. These microorganisms are commonly employed in ecotoxicological assays due to their high sensitivity to water quality changes, serving as reliable bioindicators of toxic contaminants. Effective toxicity reduction following treatment is critical for validating the HDC process, confirming that no harmful by-products are released into aquatic ecosystems [7,66,67]. Such assessments are fundamental for the development of environmentally safe and sustainable remediation technologies.

Taking into account the above, Wu et al. [36] tested the toxicity of original and treated DFC solutions by HDC on *dp*-Pd(1.7)/CeO_2_ for 2 h by an acute immobilization test of *Daphnia magna*. They observed that inhibition increased with higher DFC concentrations, confirming *daphnids* as a suitable model for assessing DFC’s acute toxicity. The 48 h EC_50_ value for DFC on the *daphnids* was determined to be 21.5% ([DFC] = 0.13 mM). For DFC solutions treated with catalytic HDC using *dp*-Pd(1.7)/CeO_2_, inhibition rates also increased with higher concentrations, but were significantly lower compared to the untreated solution. Specifically, treated solutions with 25% and 37.5% dilution showed inhibition rates of about 20% and 60%, respectively, indicating reduced toxicity. The 48 h EC_50_ for the treated solution was 35.1%, which is significantly higher than that of the untreated DFC solution. In this way, the toxicity test indicated that the DFC solution treated by HDC led to markedly decreased toxicity to *Daphnia magna*. The findings of this study allow us to see the HDC process as an effective tool in the abatement of DFC pollution in water.

Likewise, the ecotoxicity of DFC and the samples from the HDC reactions carried out by Nieto-Sandoval et al. [51] were assessed using the Microtox toxicity test (ISO11348–3, 1998) with the marine bacterium being *Vibrio fischeri*. The initial DFC solution, with a concentration of 20 mg·L^−1^, showed a high ecotoxicity (TUs (toxicity units) = 3.2) and an EC_50_ of 6.2 mg·L^−1^, which is lower than the values previously reported [16,68]. The high ecotoxicity of DFC contrasts with the lower ecotoxicity of other non-antibiotic pharmaceuticals [16,69], making DFC notable for its environmental persistence and toxicity [70]. After the HDC of DFC, the ecotoxicity of the initial DFC solution was reduced, leading to values below 10% of the initial TUs in 10 min of reaction, achieving negligible ecotoxicity values after 20 min of reaction. This behavior was attributed to the fast conversion of DFC in APA. These results indicate that the HDC process is a safe and environmentally friendly technology for removing DFC, even when it is mixed with other chlorinated micropollutants (such as CAP, SRT, and TCL)[52].

The comprehensive toxicity evaluations of post-treated DFC solutions underscore the importance of assessing hydrodechlorination performance in real, environmentally relevant water matrices to validate the practical applicability of these treatment methods.

## 5. HDC of DFC in Real Environmentally Relevant Water Matrices

The HDC of DFC is a pivotal technology for treating real water matrices due to its high effectiveness in reducing ecotoxicity and eliminating hazardous compounds. Unlike oxidative methods, which can produce toxic by-products and increase overall ecotoxicity [71,72], HDC directly addresses chlorinated compounds, which are primarily responsible for toxicity in wastewater. This process ensures efficient removal of DFC while minimizing environmental impact by producing effluents with significantly reduced ecotoxicity. Therefore, HDC represents an effective and environmentally friendly solution for treating DFC-contaminated wastewater, contributing to the protection of aquatic ecosystems and public health [5,10,73]. Most studies have used deionized water to assess processes, which is useful for initial evaluations but not reflective of real conditions.

Taking into account the aforementioned, Nieto-Sandoval et al. [51] complemented their research by testing the efficacy of the catalytic system (Pd/Al_2_O_3_) for the HDC of DFC in more complex environments. The system has been evaluated in WWTP effluent, hospital wastewater, and surface water spiked with DFC (0.068 mM). In their later research [52], the same catalyst was tested in mineral and tap water with a DFC concentration of 3 mg·L^−1^. All these mentioned water matrices are key sources of pharmaceuticals [74], and DFC is commonly found in these waters [75]. The main physicochemical parameters of these water matrices are shown in Table 3.

**Figure 3:**
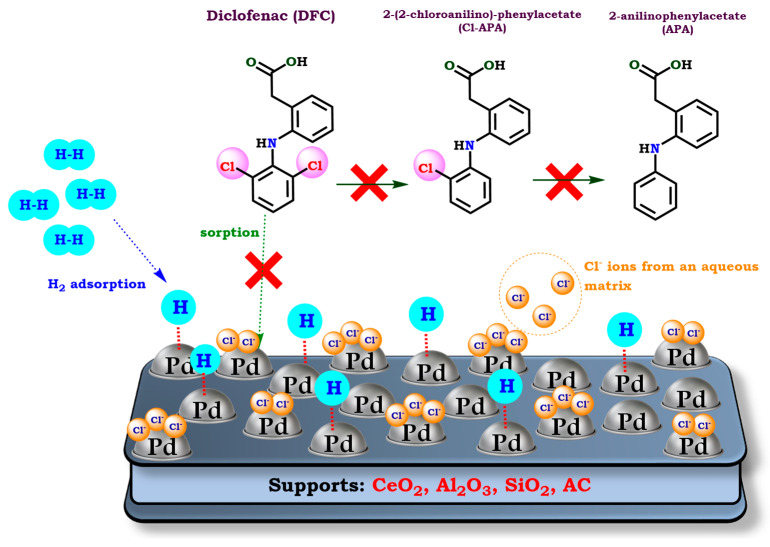
Illustration of the Pd-based catalysts poisoning during HDC of real aqueous matrices by active site blocking with Cl^−^ ions. Adapted from de Pedro et al., Ordóñez et al., and Nieto-Sandoval et al. Reprinted with permission from Ref. [51] Copyright 2018 Elsevier. Ref. [52] Copyright 2019 Elsevier. Ref. [64] Copyright 2010 Elsevier. Ref. [76] Copyright 2011 Elsevier.

The measurements in each one of these parameters are crucial because some of them, such as Cl^−^ and (SO_4_)^2−^ levels [64,76], could be directly related to the poisoning of the catalysts employed during HDC. Thus, it was observed that the presence of co-existing substances significantly impacted catalytic activity. The reduced activity in complex matrices was attributed to catalyst poisoning by high salt concentrations, particularly Cl^−^ ions, which could interact with the catalyst occupying active sites that are required for HDC (Figure 3) [64,76]. WWTP effluent had higher salt levels and lower catalytic performance compared to tap water and surface water. Despite similar carbon content in the catalyst across all matrices, hospital wastewater showed the most significant inhibition, likely due to salt poisoning rather than fouling [51]. Nevertheless, the fastest removal rate of DFC was observed in mineral water. This fact was attributed to the significantly lower chloride and total organic carbon (TOC) contents [52]. Finally, these results demonstrated that HDC is a fast and environmentally friendly technology for the removal of chlorinated micropollutants in water treatment.

In a similar fashion, De Corte et al. [26] evaluated the bio-Pd/Au NPs for the HDC of DFC from a hospital WWTP. For this purpose, they sampled the hospital WWTP effluent, and it was then treated starting with mechanical solid removal using a grid screen, followed by treatment in a conventional activated sludge (CAS) system in two aeration tanks. The aerated water was then clarified, and part of the sludge was returned to the aeration tanks. They observed that by employing the Bio-Pd/Au NPs, 43.8 ± 0.5% of the initially DFC present was removed after 24 h. The limited catalytic activity of bio-Pd/Au NPs was attributed to the presence of iodo-compounds (such as Diatrizoate that was present in this effluent at a concentration of 97.8 µg·L^−1^), which can particularly compete with chlorinated compounds for the catalytic active sites [77]. Likewise, high concentrations of sulfate in the water (30.5 mg SO_4_^2−^·L^−1^) can also potentially inhibit the activity of the catalyst, as sulfates can be reduced to sulfides, which are known to poison Pd catalysts [51,52,64,76,78]. These findings suggested that doping bio-Pd NPs with Au^0^ could be a promising strategy for the reductive treatment of hospital WWTP effluents with important concentrations of chlorinated compounds.

Nieto-Sandoval et al. [57] subsequently evaluated the versatility of the Pd/CMR system for continuous-flow HDC; for this purpose, a tap water matrix was employed as a representative effluent for the implementation of HDC in drinking water treatment applications. The Pd/CMR system demonstrated high effectiveness and long-term stability for the HDC of DFC, maintaining catalytic performance over more than 200 h of continuous operation in deionized water. However, for practical applications, this technology is envisioned as a polishing step in the final stages of DWTPs, where the water matrix is considerably more complex. Although primary treatment steps would substantially reduce pathogen load, organic matter, and suspended solids prior to HDC, the composition of real water matrices differs significantly from that of deionized water.

To evaluate the influence of such complexity on catalytic behavior, tap water was employed as the reaction matrix for an additional 25 h of continuous operation. The concentrations of DFC, Cl-APA, and APA remained constant throughout the 25 h test, confirming the remarkable stability of the catalytic membrane system, which remained active for over 220 h in total. DFC conversion was maintained at approximately 60%, comparable to that obtained in deionized water. This outcome is particularly relevant, as the presence of organic matter in real water matrices is often associated with catalyst fouling [64]. Likewise, the presence of inorganic carbon and dissolved salts did not negatively impact the catalytic performance, suggesting negligible competitive adsorption effects. In fact, the interaction of reaction-generated chloride ions with dissolved species may contribute to slight improvements in HDC activity [52].

All the findings from studies conducted in real, environmentally relevant water matrices highlight both the challenges and opportunities for the HDC of DFC, motivating the exploration of emergent biocatalytic and electrocatalytic approaches for more efficient and sustainable removal.

## 6. Emergent Approaches for HDC: Biocatalytic and Electrocatalytic Treatments for DFC Removal

To build upon the insights gained from conventional HDC systems, this section explores the complementary roles of biocatalytic and electrocatalytic approaches in advancing DFC removal strategies. Table 4 presents a horizontal comparison of the three main HDC modalities, and some of the studies carried out by using these approaches are subsequently described.

### 6.1. Biocatalytic Hydrodechlorination Processes

As discussed in previous sections, Pd-based catalysts have been widely employed for the treatment of different water sources with considerable concentrations of APIs, including NSAIDs such as CAP, TCL, SRT, and DFC [10,16,17]. Nanosized Pd catalysts are particularly promising because of their high catalytic activity and large surface-to-volume ratio. Typically, Pd catalysts are synthesized chemically and immobilized on carriers like silica to prevent agglomeration and facilitate recycling [79]. The production of Pd NPs via microbial reduction is considered both promising and environmentally friendly, as it requires fewer toxic chemicals and no stabilizers or carriers [80]. Many microorganisms can generate biogenic palladium NPs (Bio-Pd) within their cell membranes or cytoplasm [25]. Bio-Pd, a novel heterogeneous catalyst supported by bacteria, is reported to be more resistant to agglomeration and growth than NPs supported on traditional materials like activated carbon [81]. Furthermore, Bio-Pd offers advantages in terms of ease of recycling and stability over homogeneous Pd catalysts [81,82]. Nevertheless, Bio-Pds have been shown to catalyze the dehalogenation of some environmental contaminants, but they are not effective in catalyzing the degradation of other significant recalcitrant halogenated compounds.

In order to overcome the aforementioned problem, De Corte et al. [55] carried out a novel biological production of bimetallic Pd/Au NPs. These catalytic materials were evaluated for their ability to dechlorinate DFC. When aqueous Pd(II) and Au(III) ions, both at concentrations of 50 mg·L^−1^, were simultaneously reduced by *Shewanella oneidensis* in the presence of H_2_, the resulting cell-associated bimetallic NPs (bio-Pd/Au) successfully dehalogenated 78% of the initial Diclofenac concentration ([DFC]_0_ = 20 mg·L^−1^) after 24 h. In contrast, no dehalogenation was observed with either monometallic bio-Pd or bio-Au. Based on the results obtained from the different characterization techniques employed to study these catalysts (Synchrotron X-ray Diffraction (SXRD), TEM, and X-ray absorption spectroscopy (XAS)), it was possible to indicate that the concurrent reduction of Pd and Au supported on *S. oneidensis* cells led to the formation of a distinctive bimetallic crystalline structure.

In a later study, De Corte et al. [26] again evaluated the catalytic performance of bio-Pd/Au NPs during the HDC of DFC, confirming that bio-Pd/Au NPs are more efficient than bio-Pd NPs in the removal of DFC from aqueous solutions at neutral pH, with hydrogen gas (H_2_) serving as the electron donor, achieving a first-order decay rate constant of 0.078 ± 0.009 h^−1^. A disproportionate increase in catalytic activity was noted for bio-Pd/Au as the Pd content increased. In contrast, altering the Au content led to the highest catalytic efficiency at a Pd/Au mass ratio of 50:1, corresponding to a first-order decay rate of 0.52 ± 0.02 h^−1^. Finally, studies by Corte et al. [26,55] highlight that the catalytic activity and potential environmental benefits of biosupported Pd catalysts can be enhanced through coprecipitation with Au.

Quan et al. [24] synthesized biogenic nanopalladium (Bio-Pd) by using anaerobic granular sludge (AGS) to create a composite material (Pd-AGS) for the HDC of DFC by using various electron donors. The results indicated that hydrogen was the most effective electron donor for Pd-AGS, followed by formate, glucose, and acetate. Pd-AGS demonstrated the capability to generate effective hydrogen/electron donors from organic compounds through microbial metabolism, which activated Pd. More than 96% of DFC ([DFC]_0_ = 20 mg·L^−1^) was converted through HDC reaction within 90 min using Pd-AGS, achieving a maximum specific activity (*K_obs_*) of 1.53 L·g^−1^·min^−1^ at a Pd loading of 3.0 wt% in the presence of H_2_. Pd-AGS maintained high catalytic activity in phosphorus buffer solution or Na_2_SO_4_ (25 mM) at pH 7.0–7.5 but lost activity in Na_2_CO_3_ (40 mM) or NaOH (40 mM) solutions. Compared to free Pd NPs, Pd-AGS showed greater resistance to deactivation by Cl^−^ and (SO_4_)^2−^. Furthermore, Pd-AGS was capable of reducing both DFC and nitrate simultaneously with high nitrogen selectivity. As a novel form of Pd catalyst integrated with AGS, Pd-AGS showed potential for the HDC of chlorinated organic compounds in polluted water; nevertheless, the slower kinetics and variability in microbial activity can limit practical application.

**Figure 4:**
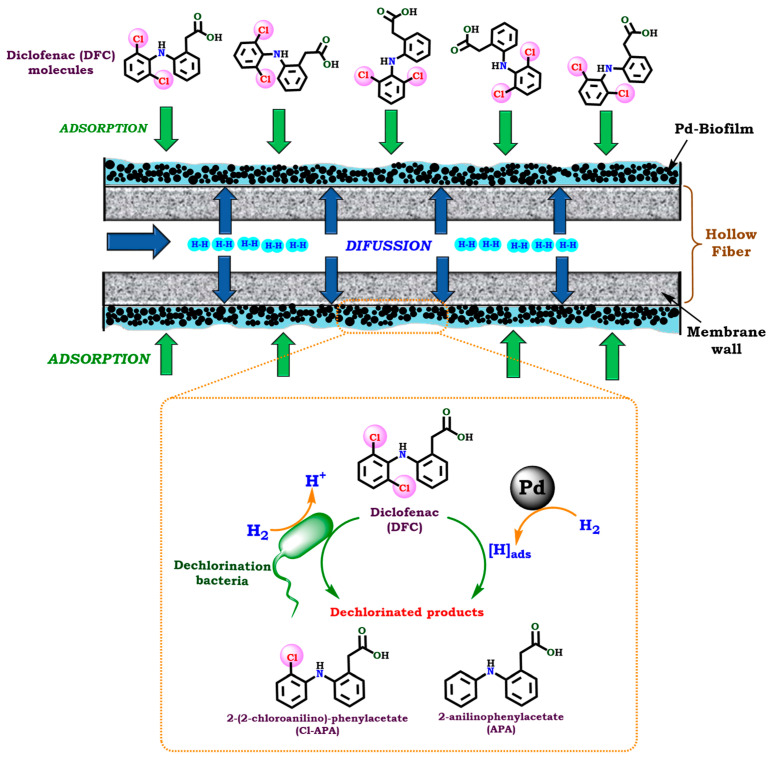
Illustration of the DFC removal by coupling Pd catalytic and microbiological processes through a H_2-_based membrane biofilm reactor (Pd-MBfR). Reprinted with permission from Ref. [27]. Copyright 2022 Elsevier.

Recently, Liu et al. [27] developed a hydrogen-based membrane biofilm reactor (H_2_-MBfR) incorporating in situ generated well-dispersed Pd NPs. This system, denoted as Pd-MBfR, was then applied in the HDC of DFC. Batch experiments revealed that the initial concentration of DFC significantly influenced both the rate and extent of its removal, while the presence of nitrate (NO_3_^−^) adversely affected the dechlorination process. Under optimized conditions, 67% removal of 0.5 mg·L^−1^ DFC and 99% removal of 10 mg·L^−1^ NO_3_^−^-N were achieved within 90 min, with a maximum DFC removal of 97% obtained in the absence of nitrate. During 78 days of continuous operation, the highest steady-state removal flux for DFC reached 0.0097 g·m^−2^·d^−1^. APA was identified as the principal transformation product. The primary mechanism for DFC removal was reductive dechlorination, driven by Pd NP-catalyzed hydrogenation, microbial reduction, and a synergistic interaction between biotic and abiotic pathways via direct hydrogen delivery (Figure 4). Additionally, DFC dechlorination was found to alter the microbial community structure within the biofilm, with the genus *Sporomusa* identified as a key contributor to DFC degradation. Overall, this work highlights the promising potential of H_2_-MBfR systems for the sustainable catalytic removal of emerging micropollutants such as DFC.

### 6.2. Electrocatalytic Hydrodechlorination (ECHDC) Processes

Recently, green and new cost-effective methods for removing DFC from wastewater effluents have been developed. Specifically, ECHDC has emerged as a promising strategy for the degradation of chlorinated organic pollutants, primarily due to its fast reaction rates, mild operational requirements, and minimal production of secondary contaminants [83]. In the ECHDC mechanism, water molecules (H_2_O) or hydronium ions (H_3_O^+^) undergo electrochemical reduction at the electrode surface, generating highly reactive hydrogen atoms (H*) [84,85]. These atomic hydrogen species are primarily responsible for attacking C-Cl bonds. Pd-based materials are widely recognized as efficient electrocatalysts in this context, owing to their strong capacity to produce and stabilize H* through Pd–H interactions and the formation of Pd hydride species [49,86]. However, certain challenges persist. Notably, the presence of large or aggregated Pd NPs on the electrode surface can significantly impair catalytic performance, and the high cost associated with Pd remains a major barrier to its widespread industrial use [87]. Currently, this problem has been assessed by using reduced graphene oxide (rGO) due to its high conductivity and large surface area, which enhances electrode performance by improving conductivity and metal dispersion [88].

From this perspective, Li et al. [58] prepared a Pd–Ni bimetallic electrode (PdNi/PPy-rGO/Ni foam) with low Pd loading via electrodeposition. The process was conducted at a deposition current of 7 mA and a temperature of 40 °C, using a Pd:Ni molar ratio of 5:1. The resulting electrode exhibited excellent electrocatalytic activity for DFC degradation, achieving complete dechlorination within 140 min. Moreover, the PdNi/PPy-rGO/Ni foam displayed improved dispersion of the active metal phase and a reduced average particle size of 3.3 nm, compared to 5 nm observed for the monometallic Pd/PPy-rGO/Ni electrode. The inclusion of rGO and Ni enhanced surface reaction kinetics and facilitated the generation of reactive hydrogen species (H*). In this way, DFC removal efficiencies between 83% and 100% were achieved. Overall, the PdNi/PPy-rGO/Ni foam electrode exhibited high catalytic efficiency and stability, indicating strong potential for application in the treatment of chlorinated contaminants in aquatic environments.

To achieve enhanced dispersion, reduced particle size, and improved catalytic performance of Pd, Li et al. [31] fabricated a Pd/polyaniline-reduced graphene oxide/nickel foam (Pd/PANI-rGO/NF) electrode by incorporating a conductive PANI-rGO interlayer for ECHDC of representative chlorinated pharmaceuticals. The electrode demonstrated remarkable removal efficiencies, achieving 99.3% degradation of DFC within 180 min. Morphological characterization via SEM and TEM revealed that the PANI-rGO interlayer promotes uniform Pd dispersion and particle size reduction. Complementary analyses using XPS, ultraviolet photoelectron spectroscopy (UPS), and DFT calculations indicated electron transfer from the PANI-rGO interlayer to Pd, resulting in electron-rich Pd sites. Furthermore, cyclic voltammetry (CV) measurements showed that the PANI-rGO layer enhances the generation of active adsorbed hydrogen (H*_ads_), thereby synergistically boosting the catalytic activity of Pd particles.

On the other hand, Wang et al. [30] focused their efforts on the development of electrocatalysts with low cost and high catalytic activity during ECHDC. From this scenario, they synthesized nitrogen-doped carbon microspheres (N-CMs) in situ via a facile hydrothermal carbonization process using shrimp shells as renewable sources of both carbon and nitrogen. The material obtained at a carbonization temperature of 650 °C (denoted as N-CM-650) exhibited a rough surface morphology, a high specific surface area of 90.14 m^2^.g^−1^, and significant nitrogen content. Pd NPs were subsequently immobilized on the N-CM surface via chemical reduction to fabricate a Pd/N-CM catalyst for ECHDC. The resulting amorphous Pd/N-CM-650 catalyst demonstrated enhanced electron transfer kinetics and high electrocatalytic activity. Under optimized conditions—comprising a Pd loading of 1.27 wt%, a cathodic potential of −1.2 V, and 20 mg of Pd/N-CM-650 as particle electrodes—95% DFC removal ([DFC]_0_: 25 mg·L^−1^) was achieved within 150 min, corresponding to a current efficiency of 34.4%. Qualitative analysis and toxicity assessment of the transformation products confirmed a significant reduction in DFC toxicity following dechlorination. Moreover, recycling experiments revealed excellent operational stability of the Pd/N-CM-650 electrode over multiple cycles.

Likewise, Wang et al. [32] synthesized carbon microsphere (CM) particle electrodes from readily available duckweed biomass for use in ECHDC. The surface morphology and chemical properties of the CMs were finely tuned by varying the pyrolysis temperature. Pyrolysis at 650 °C yielded carbon microspheres (CM-650) characterized by a high specific surface area, enriched heteroatom content (notably nitrogen and phosphorus), and excellent electrical conductivity. Subsequent loading of Ru onto CM-650 produced the Ru/CM-650 particle electrode, which, when incorporated into a three-dimensional (3D) electrochemical reactor, demonstrated superior electrocatalytic performance in the ECHDC of DFC, achieving over 90% DFC removal within 150 min. Additionally, the Ru/CM-650 catalyst exhibited remarkable stability upon repeated use, an effect attributed to the synergistic interactions between nitrogen and phosphorus heteroatoms that enhance catalyst durability.

Comparative analysis reveals that while conventional HDC systems remain the benchmark for activity and reproducibility, biocatalytic and electrocatalytic variants offer alternative routes aligned with sustainability and operational simplicity. Discrepancies in reported performance often result from differences in catalyst preparation, pollutant concentration, and reactor design.

In closing, integrating lessons from all three HDC modalities may lead to hybrid or sequential treatment systems that maximize DFC removal efficiency while minimizing cost and energy requirements. The following section summarizes the key findings of this review and outlines future research priorities.

## 7. Conclusions and Outlook

This review comprehensively addresses recent advancements in the HDC of DFC, focusing on emergent biocatalytic and electrocatalytic treatment approaches. Given the persistent and widespread occurrence of DFC in aquatic environments, the development of efficient and sustainable remediation technologies remains imperative.

Regarding biocatalytic HDC processes, they have demonstrated significant potential for the removal of DFC through the microbial synthesis of palladium NPs (Bio-Pd). These biogenic catalysts, produced by microorganisms such as *Shewanella oneidensis* and anaerobic granular sludge, offer advantages over chemically synthesized Pd NPs, including enhanced stability against agglomeration and ease of recovery. The formation of bimetallic Bio-Pd/Au NPs has been shown to markedly improve catalytic efficiency and selectivity in DFC, attributed to the unique bimetallic crystalline structures arising from concurrent Pd and Au reduction. Moreover, integrated systems such as H_2_-MBfRs with in situ generation of Pd NPs enable synergistic biotic and abiotic pathways, resulting in high removal rates and the modulation of microbial communities toward efficient DFC degraders.

On the other hand, ECHDC has emerged as a promising, green, and cost-effective approach for the degradation of chlorinated pharmaceuticals like DFC. Pd-based electrocatalysts are widely recognized for their strong ability to generate and stabilize reactive hydrogen species (H*) necessary for C–Cl bond cleavage. Recent advances have focused on enhancing catalyst dispersion, reducing Pd particle size, and lowering Pd loading through the use of composite materials such as PdNi/PPy-rGO/Ni foam and Pd/PANI-rGO/NF electrodes. These electrodes exhibit superior catalytic activity, stability, and electron transfer capabilities, leading to high DFC removal efficiencies. Additionally, biomass-derived carbon materials doped with heteroatoms and loaded with Pd or Ru NPs offer sustainable alternatives with excellent catalytic performance and operational durability, reinforcing the potential for scalable applications in wastewater treatment.

Despite significant progress, several challenges remain in advancing biocatalytic and electrocatalytic HDC technologies toward practical, large-scale applications. Biocatalytic systems benefit from environmental friendliness and unique microbial–catalyst interactions; however, their catalytic efficiency and selectivity can be limited by the intrinsic properties of bio-Pd NPs and potential mass transfer limitations in complex matrices. Further research should explore the optimization of bimetallic nanoparticle composition, synthesis conditions, and reactor design to enhance catalytic activity and long-term stability. Additionally, in-depth studies on microbial community dynamics and the fate of transformation products are essential for assessing environmental risks and maximizing biodegradation pathways. On the electrocatalytic front, although innovative electrode materials with reduced Pd content and improved dispersion have been developed, cost remains a barrier for widespread implementation. Future efforts should prioritize the development of earth-abundant, non-noble metal alternatives or hybrid catalysts that maintain high activity and selectivity. Moreover, reactor configurations that optimize mass transport, electron transfer, and catalyst regeneration could significantly improve efficiency. Integrating both biocatalytic and electrocatalytic approaches within hybrid treatment systems may offer synergistic benefits, combining the high specificity of biocatalysts with the rapid kinetics of electrocatalysis. Finally, comprehensive techno-economic and life-cycle assessments are needed to guide the translation of laboratory-scale findings into real-world wastewater treatment solutions, addressing regulatory, operational, and environmental sustainability aspects.

## Figures and Tables

**Table 1:** Catalytic systems and reaction conditions employed for DFC removal by the HDC process.

Catalysts					Reaction Conditions	DFC Conv. (%)	Ref.
Name	Preparation Method	Particle Size ** (nm)	Metal Dispersion ** (%)	S_BET_ (m^2^·g^−1^)			
*dp*-Pd(1.8)/SiO_2_	*dp:* Deposition–precipitation	9.6	---	---	**Catalyst amount:** 20 mg **[DFC]_0_:** 0.06–0.24 mM **pH of DFC solution:** 9.0 **Hydrogen donor:** H_2_. **H_2_ flow:** 250 mL·min^−1^ **Stirring:** 1400 rpm **Temperature:** 25 °C	Negligible	[36]
*dp*-Pd(1.8)/AC		9.2	---	---		29	
*dp*-Pd(1.9)/Al_2_O_3_		6.2	---	---		89	
*dp*-Pd(1.7)/CeO_2_		1.3	86	45		98	
*im*-Pd(1.8)/CeO_2_	*im*: Impregnation	5.2	21.6	66		76	
Au–Pd core–shell NPs	Precipitation between H_2_PdCl_4_ and Au NPs	8.0	---	---	**Catalyst amount:** 0.56 mg **[DFC]_0_:** 30 mg·L^−1^ **pH of DFC solution:** 7.0 **Hydrogen donor:** H_2_ **H_2_ flow:** --- **Stirring:** --- **Temperature:** 25 °C	≈100	[50]
Pd/Al_2_O_3_ *	---	---	---	270	**Catalyst load:** 0.5 g·L^−1^ **[DFC]_0_:** 68 μM **pH of DFC solution:** 6.9 **Hydrogen donor:** H_2_ **H_2_ flow:** 50 N·mL·min^−1^ **Stirring:** 900 rpm **Temperature:** 25 °C	100	[51]
					**Catalyst load:** 0.25 g.L^−1^ **[DFC]_0_:** 3 mg·L^−1^ **pH of DFC solution:** 7.0 **H_2_ flow:** 50 N·mL·min^−1^ **Hydrogen donor:** H_2_ **Stirring:** 900 rpm **Temperature:** 17–35 °C	100	[52]
Pd/SiO_2_(s)	(s)*:* Ion exchange between hydroxyl group of SiO_2_ and [Pd(NH_3_)_4_](NO_3_)_2_	1.6 ± 0.1	86	240 ± 5	**Catalyst amount:** 0.1 g **[DFC]_0_:** 240 μM **pH of DFC solution:** --- **H_2_ flow:** 1 mL·min^−1^ **Hydrogen donor:** H_2_ **Stirring:** --- **Temperature:** 25 °C	40	[53]
Pd/SiO_2_(bg) Pd/SiO_2_(bim)	Incipient wetness impregnation by using two different precursors: (bg): (CH_3_COO)_2_Pd and (bim): PdCl_2_	3.4 ± 0.2 2.9 ± 0.15	11 28	240 ± 5 240 ± 5		80 100	
PdSiBEA	Two-step post-synthesis	8.0	---	393 ± 39	**Catalyst load:** --- **[DFC]_0_:** 240 μM. **pH of DFC solution:** --- **Hydrogen donor:** H_2_ **H_2_ flow:** --- **Stirring:** --- **Temperature:** 30 °C	99	[54]
PdHAlBEA	Wet impregnation	9.0	---	377 ± 37		88	
Pd/SiO_2_	Ion exchange between hydroxyl group of SiO_2_ and [Pd(NH3)_4_](NO_3_)_2_	2.0	---	240 ± 24		65	
Pd/Al_2_O_3_	Incipient wetness impregnation	6.0	---	213 ± 21		33	
Bio-Pd/Au	Hosted in *Shewanella oneidensis*	11 ± 13.65	---	---	**Catalyst load:** 50 mg·L^−1^ **[DFC]_0_:** 20 mg·L^−1^ **pH of DFC solution:** 7.0 **Hydrogen donor:** H_2_ **H_2_ pressure:** 1 bar **Stirring:** 100 rpm **Temperature:** 25 °C	78	[55]
Bio-Pd/Au	Hosted in *Shewanella oneidensis*	11 ± 13.65	---	---	**Catalyst load:** 50 mg·L^−1^ **[DFC]_0_:** 20 mg·L^−1^ **pH of DFC solution:** 5.0–8.0 **Hydrogen donor:** H_2_ **H_2_ pressure:** 1 bar **Stirring:** 100 rpm **Temperature:** 25 °C	43.8	[26]
Pd-AGS	Hosted in AGS	85% of particles within the size range of 0–10	---	---	**Catalyst load:** 80 mg·L^−1^ **[DFC]_0_:** 20 mg·L^−1^ **pH of DFC solution:** 7.0 **Hydrogen donor:** H_2_ **H_2_ pressure:** --- **Stirring:** 180 rpm **Temperature:** 35 °C	96.5	[24]
Raney Al–Ni *	---	---	---	---	**Catalyst load:** 1.65 g.L^−1^ **[DFC]_0_:** 0.59 g.L^−1^ **[KOH]_0_:** 9.7 g.L^−1^ **Stirring:** 750 rpm **Temperature:** 25 °C	100	[56]
Pd/CMR	Ion adsorption	6 ± 1.6	---	3	**Q_Effluent_:** 0.1 and 0.2 mL·min^−1^ **[DFC]_0_:** 100–500 µg·L^−1^ **pH of effluent:** 6.9 **H_2_ flow:** 50 N·mL·min^−1^ **Temperature:** 25 °C	58–64	[57]
Pd/PANI-rGO/NF	Electrodeposition	2.2	---	---	**[DFC]_0_:** 20 mg·L^−1^ **Anolyte:** 50 mL Na_2_SO_4_ 0.05 mol.L^−1^ **Catholyte:** 50 mL DFC mixed with Na_2_SO_4_ 0.05 mol.L^−1^ **Current density:** 1.75 mA.cm^−2^ **Temperature:** 40 °C	99.3	[31]
Ru/CM-650	Hydrothermal carbonization	150	---	193	**Anolyte:** 0.2 mol.L^−1^ H_2_SO_4_ solution **Catholyte:** 80 mL of 25 mg·L^−1^ DFC solution containing 0.05 mol.L^−1^ Na_2_SO_4_ electrolyte with Ru/CM particle electrode **[DFC]_0_:** 20 mg·L^−1^ **Cathode potential:** −1.2 V	>90	[32]

* Commercial catalysts; ** Values calculated from TEM.

**Table 2:** Kinetic parameters of the HDC of DFC by using different catalysts.

Catalyst	T (°C)	$k_{1}$ (min^−1^)	$k_{2}$ (min^−1^)	$k_{3}$ (min^−1^)	$\frac{k_{2}}{k_{1}}$	$R^{2}$	Kinetic Model	Ref.
*dp*-Pd(1.9)/Al_2_O_3_	25	0.009	0.027	0.030	3.0	0.996	Langmuir–Hinshelwood	[36]
*dp*-Pd(0.45)/CeO_2_		0.010	0.020	0.030	2.0	0.998		
*dp*-Pd(0.9)/CeO_2_		0.017	0.045	0.034	2.7	0.998		
*dp*-Pd(1.7)/CeO_2_		0.025	0.108	0.049	4.3	0.997		
*dp*-Pd(2.6)/CeO_2_		0.028	0.132	0.053	4.7	0.995		
Au-Pd core–shell NPs	25	0.027	---	---	---	0.994	First order	[50]
		0.014	0.038	---	2.7	0.997	Second order	
Pd/Al_2_O_3_ *	17	0.107	0.069	0.286	0.6	0.949	Pseudo-first order	[51]
	25	0.175	0.145	0.394	0.8	0.957		
	35	0.301	0.240	0.734	0.8	0.944		
Pd/SiBEA	30	0.190	---	---	---	0.993	First order	[54]
Pd/HAlBEA		0.110	---	---	---	0.986		
Pd/SiO_2_		0.020	---	---	---	0.948		
Pd/Al_2_O_3_		0.090	---	---	---	0.997		

* Commercial catalysts.

**Table 3:** Main physicochemical parameters of the real aqueous matrix.

Matrices	pH	TOC (mg·L^−1^)	IC (mg·L^−1^)	Conductivity (μS.cm^−1^)	Cl^−^ (mg·L^−1^)	(SO_4_)^2-^ (mg·L^−1^)	Ref.
WWTP effluent	7.1	2.6	19.7	462	75.0	39.5	[51]
Surface water	7.0	2.7	14.9	200	14.1	11.2	
Hospital wastewater	8.6	110	63	1185	216.8	33.8	
Mineral water	7.1	0.3	3.5	41	0.35	---	[52,57]
Tap water	7.2	2.5	2.9	67	8.7	---	
Hospital WWTP	7.4	---	---	---	---	30.5	[26]

**Table 4:** Horizontal comparison of the three main HDC modalities.

Technology	Catalyst Type	Reaction Medium	Advantages	Limitations
Conventional HDC	Pd/SiO_2_, Pd/AC, Pd/Al_2_O_3,_ Pd/CeO_2_, Pd/zeolite	H_2_ in aqueous phase	High removal efficiency, well-studied	Requires external H_2_, catalyst deactivation
Biocatalytic HDC	Bio-Pd/AGS, Bio-Pd/MBfR, Bio-Pd/Au NPs	Anaerobic/neutral media	Low-energy, self-regenerating systems	Lower rates, biomass variability
ECHDC	Pd/N-CMs, Pd/PANI-rGO	Electrochemical cell	No H_2_ needed, high control of conditions	Requires a power source, electrode degradation

## Data Availability

No data was used for the research described in the article.

## Abbreviations

The following abbreviations are used in this manuscript:

Hydrodechlorination	HDC
Diclofenac	DFC
Active pharmaceutical ingredients	APIs
Non-steroidal anti-inflammatory drugs	NSAIDs
Wastewater treatment plants	WWTPs
Advanced oxidation processes	AOPS
Acetaminophen	ACE
Ciprofloxacin	CPFX
Nanoparticles	NPs
Extracellular polymeric substance	EPS
Membrane biofilm reactors	MBfRs
Electrocatalytic hydrodechlorination	ECHDC
Chloramphenicol	CAP
Triclosan	TCL
Sertraline	SRT
Ecological structure–activity relationships	ECOSARs
Density functional theory X-ray diffraction	DFT XRD
Transmission electron microscopy	TEM
X-ray photoelectron spectroscopy	XPS
Scanning electron microscopy	SEM
High-resolution TEM	HRTEM
Total reflection X-ray fluorescence	TXRF
2-anilinophenylacetate	APA
2-cyclohexylaminophenylacetate	CPA
Drinking water treatment plants	DWTPs
Temperature-programmed hydride decomposition	TPHD
Hydrodehalogenation	HDH
Catalytic membrane reactor	CMR
Inductively coupled plasma mass spectrometry	ICP-MS
Thermogravimetric analysis	TGA
Turnover frequency	TOF
(2-(2-chloroanilino)-phenylacetate)	Cl-APA
Toxicity units	TUs
Total organic carbon	TOC
Conventional activated sludge	CAS
Synchrotron X-ray diffraction	SXRD
X-ray absorption spectroscopy	XAS
Anaerobic granular sludge	AGS
Reduced graphene oxide	rGO
Polyaniline-reduced graphene oxide/nickel foam	PANI-rGO/NF
Ultraviolet photoelectron spectroscopy	UPS
Cyclic voltammetry	CV
Carbon microspheres	CMs
